# Hierarchical folding of multiple sequence alignments for the prediction of structures and RNA-RNA interactions

**DOI:** 10.1186/1748-7188-5-22

**Published:** 2010-05-21

**Authors:** Stefan E Seemann, Andreas S Richter, Jan Gorodkin, Rolf Backofen

**Affiliations:** 1 Center for non-coding RNA in Technology and Health, IBHV, University of Copenhagen, Grønnegårdsvej 3, Frederiksberg C, 1870, Denmark; 2 Bioinformatics Group, University of Freiburg, Georges-Köhler-Allee 106, Freiburg, 79110, Germany

## Abstract

**Background:**

Many regulatory non-coding RNAs (ncRNAs) function through complementary binding with mRNAs or other ncRNAs, *e.g*., microRNAs, snoRNAs and bacterial sRNAs. Predicting these RNA interactions is essential for functional studies of putative ncRNAs or for the design of artificial RNAs. Many ncRNAs show clear signs of undergoing compensating base changes over evolutionary time. Here, we postulate that a non-negligible part of the existing RNA-RNA interactions contain preserved but covarying patterns of interactions.

**Methods:**

We present a novel method that takes compensating base changes across the binding sites into account. The algorithm works in two steps on two pre-generated multiple alignments. In the first step, individual base pairs with high reliability are found using the PETfold algorithm, which includes evolutionary and thermodynamic properties. In step two (where high reliability base pairs from step one are constrained as unpaired), the principle of cofolding is combined with hierarchical folding. The final prediction of *intra*- and *inter*-molecular base pairs consists of the reliabilities computed from the constrained expected accuracy scoring, which is an extended version of that used for individual multiple alignments.

**Results:**

We derived a rather extensive algorithm. One of the advantages of our approach (in contrast to other RNA-RNA interaction prediction methods) is the application of covariance detection and prediction of pseudoknots between *intra*- and *inter*-molecular base pairs. As a proof of concept, we show an example and discuss the strengths and weaknesses of the approach.

## Background

Predicting RNA-RNA interactions is a rapidly growing area within RNA bioinformatics and is essential for the process of assigning function to known as well as *de novo *predicted non-coding RNAs (ncRNAs) such as those identified in *in silico *screens for RNA structures [1-7]. This candidate information along with the data generated from deep sequencing analyses emphasise the need to predict RNA-RNA interactions. In part, this is because there currently is no high-throughput method available for the reliable analysis of RNA-RNA interactions; however, computational prediction of RNA-RNA interactions is also essential for the identification of putative targets of known and *de novo *predicted ncRNAs. With the main exception of microRNA target prediction, the current approaches essentially evaluate the stabilities of the common complexes between ncRNAs and target RNAs by computing the overall free energy using two major strategies (see, *e.g*., [8] for a recent review).

The first strategy, represented through the implementations of RNAup[9] and IntaRNA[10], uses pre-calculated values for all possible regions of interaction to determine the energy required to make that site accessible (called the ED-value for the energy difference). The ED-value is then used to calculate a combined energy of the energy given by the duplex formed by the two interaction regions and the ED-values of both interaction regions. RNAup has a complexity of *O*(*n*^3 ^+ *nw*^5^), whereas IntaRNA has a complexity of *O*(*n*^2^), which makes it fast enough to be used in genome-wide screens. Both methods are able to predict complex interactions, like kissing hairpins, *as long as *the interaction is restricted to one region. However, there are well-known examples where several interaction sites were found, especially for longer ncRNAs. A prominent example is the interaction between OxyS and *fhlA *shown in [11].

The second strategy for RNA-RNA interaction predictions is usually handled with a class of approaches that simultaneously predict a common structure for both RNAs including their interaction. Some of the first approaches, *e.g*., pairfold[12], RNAcofold[13] and the method presented by Dirks *et al*. as part of the NUpack package [14], concatenate the two sequences using a special linker character. Then, a modified version of the usual RNA folding methods (like Mfold[15] and RNAfold[16]) is applied to cope with the linker symbol to predict the correct energies. Otherwise, a loop containing the linker symbol would be treated like a hairpin or internal loop, leading to incorrect energy values.

The main disadvantage of the concatenation approach is that the set of candidate joint structures becomes restricted. For this reason, double kissing hairpin interactions (like in OxyS-*fhlA*) cannot be considered. However, alternative (but also most resource demanding) methods have been introduced and extend the class of allowed joint structures. The IRIS tool [17] allowed several kissing hairpins using a maximum number of base pair energy model. Then, Alkan *et al*. [18] presented a more realistic energy model and showed the NP-completeness of an unrestricted model. Both approaches predict structures with minimum free energy.

A more stable approach is to consider the partition function because it allows the calculation of interaction probabilities and melting temperatures. This problem was solved independently by Chitsaz *et al*. [19] and Huang *et al*. [20]. In [21], hybrid probabilities were calculated. These approaches have high time complexities of *O*(*n*^6^), which makes them infeasible for genome-wide applications. Methods to reduce the complexity range from approximation approaches [22,23] to sparsification of the dynamical programming matrix [24].

Here, we present an algorithm for the prediction of RNA-RNA interactions in existing multiple alignments of RNA sequences. Its rationale is based on the assumption that a non-negligible amount of the RNA-RNA interactions contain compensatory base changes across the binding sites. The algorithm presented herein is an extension of the PETfold algorithm [25] and makes further use of the principles from RNAcofold [13] and computational strategies for hierarchical folding, *e.g*. [26,27]. The latter approach was chosen due to the high computational costs of pseudoknot searches.

## Algorithm

The main idea of the introduced method is to use a hierarchical approach to predict an interaction by predicting reliable base pairs within a ncRNA and a mRNA (or another ncRNA), which is followed by prediction of reliable base pairs in the combined sequence. Via this approach, we are able to predict combined pseudoknotted structures, like kissing hairpins, that would be missed otherwise. In both steps, we apply a combined scoring method that predicts consensus base pairs from an alignment using evolutionary conservation and thermodynamic stability information.

The scoring for the first step is according to the standard PETfold approach, where we use thresholds for reliable base pairs that have been identified according to training on more than 30 verified interactions in bacteria, which is described later. For the second step, we define a constrained version of the PETfold scoring scheme.

Throughout this paper, we consider the concatenation of the two alignments and subsequently (in the base pair prediction process) the concatenation of the corresponding structures. *σ *will denote a set of base pairs, where the substructures in each part (*e.g*., ncRNA, mRNA and the base pairs that participate in the interaction) in respective alignments are concatenated or nested (in the dot bracket notation, these substructures have alignment lengths of the ncRNA and mRNA respectively). We use (*i*, *j*) to denote a Watson-Crick or G-U wobble base pair between columns *i *and *j*. This base pair could be an *intra*-molecular pair in each of the RNA molecules (ncRNA or mRNA) or an *inter*-molecular pair that is involved in an interaction between molecules.

Depending on the context, *σ *will either be interpreted as a specific structure that implicitly defines the single-stranded positions or as a partial structure that describes an ensemble of structures. In the first case, we define the set of single-stranded positions of a sequence *s *as

In the second case, we use ℰ(*σ*) = {*σ'*|*σ' *⊇ *σ*} to denote the ensemble of all specific structures *σ' *extending *σ*. (*s*) denotes the set of nested secondary structures that are defined for the sequence *s*. We use the same notation for the consensus structures of a given multiple alignment  with *n *sequences *s*^1 ^... *s*^*n*^. In this case, a position 1 ≤ *i *≤ || refers to a column in the alignment. Furthermore, we use *s *∈  to indicate a sequence *s*^1 ^... *s*^*n *^from the alignment.

The algorithm, like PETfold is a maximum expected scoring approach that combines the evolutionary probabilities Pr^ev^[*σ*|] of a consensus structure, *σ*, given an alignment, with the thermodynamic probabilities of the associated structures in each sequence. Pr^ev^[*σ*|] is generated using the stochastic context-free grammar (SCFG) from the Pfold model [28]. The Pfold model allows the computation of the probability Pr[*σ*|, *T*, *M*] of a consensus structure *σ *given an alignment , a phylogenetic tree *T *for that alignment, and a general background model *M *for secondary structures. Because the tree *T *is calculated from the alignment , and *M *is constant, we use Pr^ev^[*σ*|] as short for Pr[*σ*|, *T*, *M*].

The (secondary structure) model itself is based on a SCFG that provides a distribution of secondary structures for a given alignment. The combined probability of an alignment  and a consensus structure *σ *is

where Pr[*σ*|*T*, *M*] is the prior distribution of secondary structures and Pr[|*T*, *σ *] is the probability of the alignment, given a known consensus structure. This is then transformed into Pr[, *σ*|*T*, *M*] by applying the Bayesian rule, and further into the posterior distribution Pr[*σ*|, *T*, *M*] of consensus structures *σ *by dividing by Pr[|*T*, *M*], which is the sum of all parse trees for an alignment  given *T *and *M*. Note that the comma sign here is just a shortcut for ∧, *i.e*. Pr[*A*, *B*] = Pr[*A *∧ *B*]. We will still use ∧ where it is appropriate.

The probability distributions themselves are formed as follows. For Pr[|*T*, *σ *], there is an independent evaluation of all base pairs and single-stranded positions:

where  is the i*th *column of , and  for the constrained folding, where ( resp.) is the constrained structure on the first (second resp.) of the two concatenated alignments. For the prior model, the probability Pr[*σ*|*T*, *M*] provides an overall distribution of the secondary structures, which is estimated from rRNA and tRNA sequences. *M *is given by the following simple SCFG:

The evolutionary model and the prior model for RNA structures used in the Pfold model are combined into a single SCFG that provides a distribution over Pr[*A*, *σ*|*T*, *M*] (see additional file 1 for details).

To model the thermodynamic probabilities, we define *σ *(*s*^*k*^, ) as the structure for the *k*-th sequence *s*^*k *^of an alignment  associated with the consensus structure *σ *of . Pr^th^[*σ *(*s*^*k*^, )|*s*^*k*^] is the corresponding thermodynamic probability as defined by McCaskill's partition function approach [29].

Using the maximum expected scoring approach, these probabilities are transformed into reliabilities in a two-step approach. Throughout the paper, (*i*) is used to denote the reliability of a single-stranded region at alignment position *i *and (*i*, *j*) the reliability of a consensus base pair (*i*, *j*), where ℓ = 1, 2 refers to Step 1 or Step 2 of the combined approach.

### Refresher: PETfold scoring

Here, we briefly recall the scoring of PETfold, which is a maximum expected accuracy scoring method. For simplicity, we will exclude a description of the scoring of single-stranded positions. However, they are scored the same way as in the original PETfold approach; for more details, see [25].

The PETfold score is the sum of the evolutionary accuracy values plus the average sum of the thermodynamic accuracy values. For the evolutionary part, we compute the expected accuracy (or overlap) EA^ev^(*σ*) of a specific consensus structure *σ *with all possible consensus structures, which are weighted according to their probabilities:(1)

Recall that Pr^ev^[*σ'*|] denotes the evolutionary probability of a structure *σ' *according to the Pfold SCFG as described above. |*σ *∩ *σ'*| is the number of base pairs that are common between *σ *and *σ' * and thus denotes the overlap between these two structures.

For the thermodynamic part, the expected accuracy EA^th^(*σ*) of *σ *with all structures for all sequences according to the thermodynamic ensembles is defined by(2)

The combined expected accuracy consists of both parts, generally weighted with 1 for the conservation portion and *β *for the thermodynamic accuracy:(3)

where *n *is the previously described number of sequences in the alignment. As shown previously [25], this final score can be calculated using the base pair reliabilities, where the combined reliability ℛ_bp_(*i*, *j*) for a base pair (*i*, *j*) is given by(4)

where (*i*, *j*, *s*) is the base pair probability of the pair (*k*, *l*) associated with columns (*i*, *j*) in sequence *s*. These reliabilities are calculated with an inside/outside algorithm and are central to the hierarchical approach presented in the following sections. The expected accuracy can then be calculated from the base pair reliabilities by(5)

The consensus structure with the maximal reliability is then calculated using a Nussinov-style algorithm [30], where the base pairs are evaluated with reliabilities.

### Step 1: Intra-molecular partial structures

We use two alignments  and  of sequences  and , where  is a ncRNA and  is its target sequence. For convenience, we adopt the convention of RNAcofold and assume that the positions in  are numbered 1 ≤ *i *≤ || and the positions in  are numbered || + 1 ≤ *i *≤ || + ||.

#### Selection of the initial structure

In the first step of the pipeline, we obtain the base pair reliabilities from Equation (4), which we denote (*i*, *j*). Using these reliabilities, the partial (constrained) structures  and  are determined independently for  and . In the following steps, let  be either  or  and *σ*^p ^be the partial structure calculated for . This is done by selecting only base pairs (*i*, *j*) with

where *δ *is a cut-off that must be ≥ 0.5 to avoid crossing structures. This is similar to the method by which consensus structures are predicted for single sequences [31] and has been shown to be more reliable for the prediction of consensus structures from alignments.

Here, however, we also have to estimate the contribution of each of the partial structures to the complete solution. Because the set of base pairs from a predicted consensus structure do not necessarily form a reasonable structure, we account for this by introducing a second threshold *γ*. High values for this threshold guarantee that each sequence used to create the consensus structure has a high likelihood and that the approximation, which we apply in the second step (as will be described by Equation (14)), is accurate.

To find the optimal value of the reliability threshold *δ*, its value is increased until the resulting ensemble of structures ℰ(*σ*^p^) that are compatible with the partial structure *σ*^p ^is probable enough in the evolutionary model, in the thermodynamic model, or in both models, which is when

Here, Pr^ev^[ℰ(*σ*^p^)|] (= Pr^ev^[ℰ(*σ*^p^)|, *T*, *M*]) is the probability of the partial structure *σ*^p ^given the alignment  in the evolutionary model *M *and tree *T*. This can be calculated from Pfold with the SCFG that combines the prior structural model with evolutionary information from the alignment (see additional file 1) as follows:(6)

The term Pr[|*T*, *M*] has already been calculated (personal communication with Bjarne Knudsen) in Pfold as the sum of all possible parse trees for an alignment , given *T*, *M*:

Here, we add the calculation of

to Pfold by summing over all possible parse trees that are compatible with *σ*.

Pr^th^[ℰ(*σ*^p^(*s*, ))|*s*] is the probability of the partial structure *σ*^p ^given a sequence *s *in the thermodynamic model. This probability can be calculated using constrained partition folding as follows:(7)

where  is the free energy of the whole ensemble (as determined by RNAfold with parameters -p -d2) and  is the free energy of the ensemble of structures ℰ(*σ*^p^(*s*, )) with the base pairs in *σ*^p^(*s*, ) as constraints, which can be calculated by RNAfold with parameters -C -p -d2.

#### Extension of constrained stems

Reliable *intra*-molecular base pairs are constrained as single-stranded in Step 2 of the algorithm because we are interested in pseudoknots of the concatenated sequence and the interactions in these induced loop regions. The drawback of this *ansatz *is that *intra*-molecular stems get instable because of intermediate unbased constraints. Thus, we may get incomplete stems. To deal with this problem, we extend the constrained stems. Inner and outer base pairs are added as long as the average reliability of the inner or outer extended stem, respectively, is larger than the threshold *δ*, and the probability of the partial structure is greater than or equal to *γ *either in the evolutionary or the thermodynamic model. That is, the average reliability of the total, extended stem has to be larger than a threshold.

**Figure 1:**
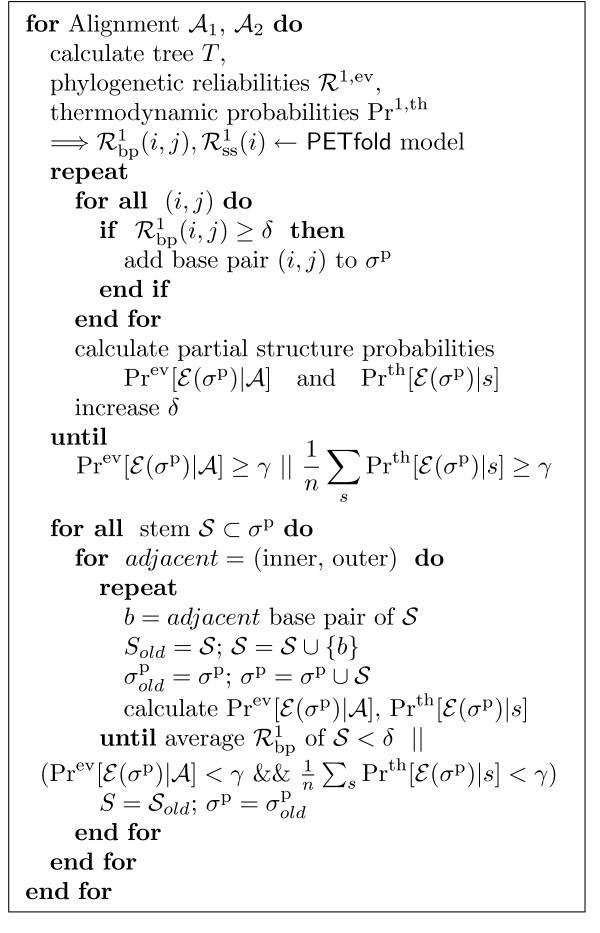
Pseudocode for Step 1.

Step 1 is summarised as pseudocode in Figure 1.

### Step 2: Constrained expected accuracy scoring

In the following, *s*_1_&*s*_2 _denote the concatenated sequences of the two sequences *s*_1_, *s*_2 _using the additional linker symbol & as done in RNAcofold. For Step 2 of the scoring, we calculate the expected accuracy of the ensemble of structures *σ *of *s*_1_&*s*_2_, which constitutes an interaction under the constraint that *σ *contains the partial reliable structures  and  of *s*_1 _and *s*_2_, respectively. Because we use the numbering convention of RNAcofold, the union  of the two partial structures  and  is the partial structure of *s*_1_&*s*_2_.

Now we have two problems to solve. On the one hand, we want to calculate the constrained accuracy given the partial structures  and , which is defined as(8)

On the other hand, we have to find a combined score for the partial structures  and , and the interaction *σ*_int _to evaluate the quality of an predicted interaction. The score must be maximal according to Equation (8).

We will demonstrate the problem and our solution for the thermodynamic folding. However, the same analysis applies to the evolutionary part, which is described later.

#### The thermodynamic part

The simplest formal solution to this problem would be to investigate directly the expected accuracy of joint structures *σ*:

where  is the expected accuracy of a structure in one sequence pair *s*_1_&*s*_2 _∈ .

However, this would require that we compute the distribution Pr^th^[*σ*|*s*_1_&*s*_2_], which can be done by a partition function approach for interacting structures. This is NP-complete in the full model [18] and even *O*(*n*^6^) in a restricted model [19,20], which is why the two-step approach is necessary. In the following, we ignore the index "th" for simplicity.

The relationship between  and EA(*σ*) is now quantified. In the following, for a structure *σ*, we use *σ*_1 _∪ *σ*_2 _∪ *σ*_int _to denote the partition of the base pairs of the first sequence, *σ*_1_, the base pairs of the second sequence, *σ*_2_, and the interacting base pairs, *σ*_int_. Furthermore, for the partial structure *σ*, we use ℰ_1_(*σ*) to denote the set of structures that extends *σ *using base pairs within the first sequence, *i.e*.,

The ensembles ℰ_1,int_(*σ*), ℰ_2,int_(*σ*) and ℰ_1,2_(*σ*) are defined analogously.

Our approach uses one simplification, namely the assumption that the reliabilities for *intra*-molecular base pairs are dominated by the *intra*-molecular folding. This is equivalent to the assumption that the two structures fold independently. We formulate this as follows:

Because *σ*_1 _and *σ*_2 _are *partial joint *structures, this can be written using the ensemble function(9)

The implication of this assumption is that the probabilities of the two structures *σ*_1 _and *σ*_2 _are merged independently into the joint probability Pr[ℰ_int_(*σ*_1 _∪ *σ*_2_)|*s*_1_&*s*_2_], see Equation (11) below. First, note that for two partial structures

by definition. Hence,

Intuitively, Pr[ℰ_1,int_(*σ*_2_)|*s*_1_&*s*_2_] should be the same as Pr[*σ*_2_|*s*_2_]. This can be derived using the total probability formula:(10)

Combining these equations we obtain the independence property:(11)

Now we will use this property to relate  to EA(*σ*). The independence property, as described in Equation (9), and the additivity of the expectation is the implication of the expected accuracy of a joint structure, which is the sum of the expected accuracy of the *intra*-molecular structures and the expected accuracy of the *inter*-molecular portion. To illustrate this, note that for any *σ*, *σ'*

by definition. Hence, by the additivity of the expectation we get

Now we can rewrite the first term  using the independence property as follows:

which is the expected accuracy of *σ*_1 _in the sequence *s*_1_. Analogously, we can do this for the second term . Thus,  is the sum of the expected accuracies in the first and the second sequences and the expected accuracy of the interaction:(12)

For the expected accuracy of the interaction(13)

we still need to define Pr[*σ*|*s*_1_&*s*_2_]. For every *σ *= *σ*_1 _∪ *σ*_2 _∪ *σ*_int_,

Thus, in principle, to calculate the expected accuracy EA^th, int ^(*σ*) for the interaction, we must sum over all structures in *σ*_1 _and *σ*_2_:

Because this is not feasible, we restrict ourselves to an ensemble of structures. Thus, instead of summing over all possible *σ*_1 _and *σ*_2_, we use the partial structures  and  that were determined in the first step and approximate EA^th,int^(*σ*) by

The second sum can now be simplified as follows:

where Equation (11') indicates the variation of the independence assumption of Equation (11) for the structure ensembles (see additional file 1). Thus, we finally have(14)

Now  is the constrained folding, where the positions covered by  and  are fixed. However, we have the problem that these structures might contain pseudoknots. Recall that the positions in  and  are fixed for folding and that we are considering all structures *σ *that contain  and are nested on . Technically, we solve the problem using the fact that the set of structures that is nested on *σ*_int _*and *compatible with  is selected by considering all structures where the positions of  are constrained as single-stranded. This implies that we use constrained cofolding via RNAcofold (parameters -C -p -d2), and the constraint ... *x*_1_*x*_1 _... & ... *x*_2_*x*_2 _..., where *x*_1 _(resp. *x*_2_) denotes a position from  (resp. ) that is constrained as single-stranded. The main difference is that the energy contributions could be slightly different, and therefore, we obtain only an approximation of the real distribution. For example, an extension of a helix in  would be evaluated as an internal loop or hairpin. Note that this is not a major problem because we are mainly interested in the *inter*-molecular base pairs between *s*_1 _and *s*_2 _in this step. However, the recursion scheme of RNAcofold could easily be adapted to use new symbols for base pair constraints and a scoring scheme that is common to hierarchical approaches of pseudoknot structure prediction, which would avoid these problems.

Finally, we can rewrite the thermodynamic accuracy as the sum of probabilities as indicated in Equation (5). As shown in Equation (12), for a base pair (*i*, *j*) ∈  (ℓ = 1, 2), we want to use the probability of the associated sequence. To avoid competition with the probabilities for the *intra*-molecular base pairs calculated from RNAcofold, we set all of these base pairs to the same probability  as described in Equation (7). For the *inter*-molecular base pairs, we use the base pair probabilities as provided by RNAcofold with constraints, which model  from the constrained cofolding. However, these raw base pair probabilities (in the following denoted by ) are calculated under the constraint of  and have therefore (to obtain the final base pair probabilities) to be multiplied by  as indicated by Equation (14). Thus, we can score each base pair as follows:(15)

where the 1 reflects the fixed reliability. However, we deviate from this scoring to weaken the independence assumption for the *intra*-molecular base pairs, which allows us to determine new *intra*-molecular base pairs from the constrained application of RNA-cofold. Thus, we score only the base pairs from the partial structures  and  with the probability in the associated sequence. In addition, to avoid competition with the probabilities for these base pairs calculated from RNAcofold, we simply set all of these base pairs to the same probability  as described in Equation (7). To summarise, given the partial consensus structures  and  for an alignment  as calculated in Step 1, the probability for a base pair (*i*, *j*) in sequence *s*_1 _&*s*_2 _∈  in the second step is:(16)

#### Single-stranded probabilities

Single-stranded probabilities are integrated in a similar way as the base pair probabilities, but with different weighting. The single-stranded probabilities are as follows:(17)

Given the structure *σ *on an alignment  with *m *columns, the set of all single-stranded positions in the consensus structure is denoted as ss(*σ*) = {*i *∉ *σ*|1 ≤ *i *≤ *m*}. Taking this into consideration, the complete version of Equation (2) is

and the evolutionary accuracy is determined similarly. The combined score is the sum of the base pair reliabilities and single-stranded reliabilities (weighted with the parameter *α*). For details, see [25].

#### The evolutionary part

The calculation for the presented thermodynamic accuracy is purely based on constrained folding. To obtain the complete constrained folding, we use the same approach for the evolutionary accuracy by applying a version of Pfold[28] that incorporates the constraints. For that purpose, the raw structural reliabilities (*i*, *j*) and (*i*) are calculated by the constrained folding with Pfold using the phylogenetic tree deduced from the concatenated alignment. As a linker, three prior-free columns are inserted between both alignments. The evolutionary reliabilities (*i*, *j*) for a base pair (*i*, *j*) and (*i*) for a single-stranded position *i *are calculated in the same manner as  in Equation (16):(18)

as well as  in Equation (17):(19)

The probabilities of the partial structures  and  are calculated as described in Equation (6). Step 2 is summarised as pseudocode in 1.

**Figure 2:**
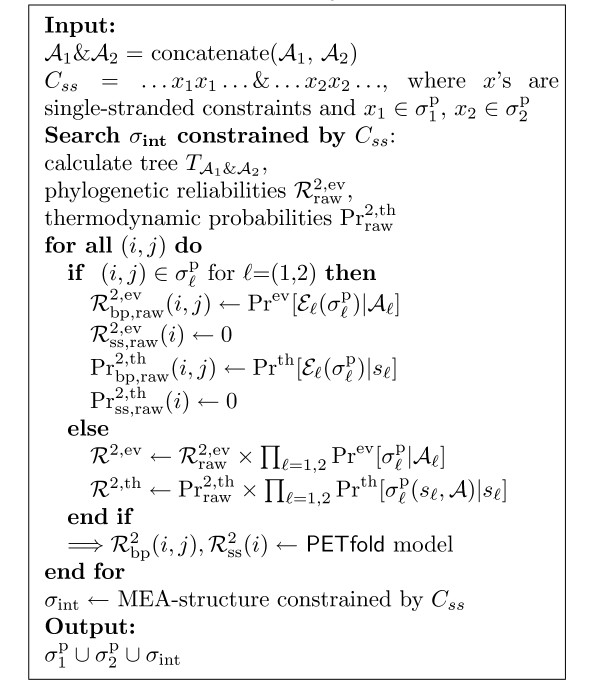
Pseudocode for Step 2.

#### The final scoring

To summarise the reliabilities, a combined structure will be determined using the Nussinov algorithm on the following reliabilities:

where  and  are defined as above,  as in Equation (16) and  as in Equation (17).

**Figure 3:**
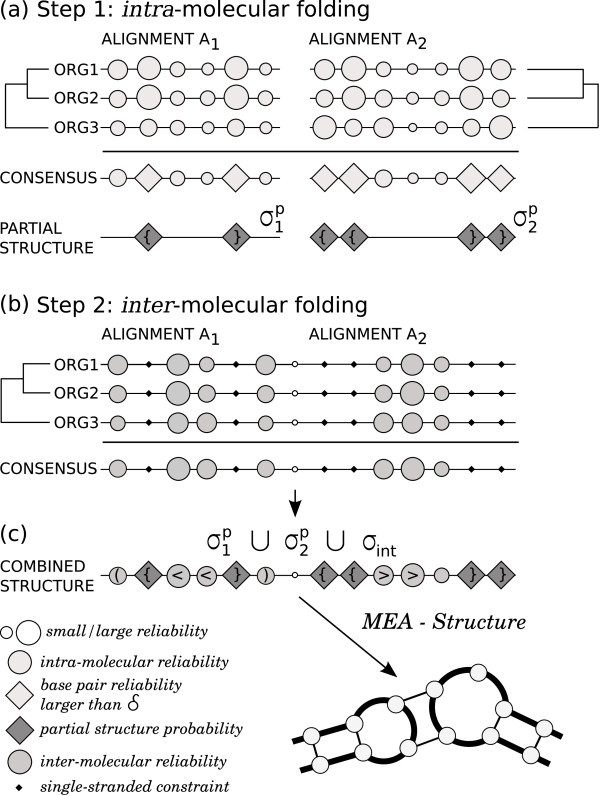
**Scoring pipeline**. The pipeline illustrates the flow of base pair probabilities during the structure scoring. The PETcofold pipeline consists of two steps: (a) *intra*-molecular folding by PETfold of both alignments and selection of a set of highly reliable base pairs that form the partial structures *σ*^p^; (b) *inter*-molecular folding by an adaptation of PETfold of the concatenated alignments using the constraints from Step 1. In the end, (c) the partial structures and constrained *inter*-molecular structures are combined to generate the joint secondary structure including pseudoknots.

Note that the base pairs in  have a weight of 0 during folding of the constrained structure to allow for pseudoknot formation. Finally, we add the base pairs in  to the constrained structure of Step 2. The flow of the structure reliabilities in the pipeline is summarised in Figure 3.

## Results and discussion

The algorithm presented herein was implemented in PETcofold (Seemann *et al*., submitted). As a proof of concept, we present an example of a bacterial sRNA-mRNA interaction. The in-depth analysis is described elsewhere (Seemann *et al*., submitted).

### Joint structure prediction of bacterial sRNA OxyS and its target mRNA fhlA

The small RNA OxyS represses the translation of the mRNA *fhlA*, which is mediated through base pairing at the ribosome binding site [11]. However, the OxyS-*fhlA *interaction involves a second binding site within the coding region of *fhlA*. Both interaction sites reside in stem loops such that OxyS and *fhlA *form a double kissing hairpin interaction.

**Figure 4:**
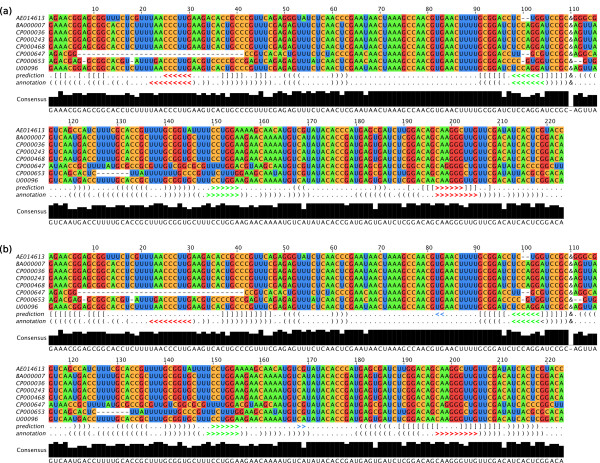
**Joint secondary structure prediction of the sRNA OxyS and its target mRNA fhlA**. The sequence alignment shows the two input alignments concatenated by the linker symbol "&", the joint secondary structure predicted by our algorithm (with *δ *= 0.9 and *γ *= 0.1 and disallowing base pairs that only occur isolated in the thermodynamic part) and the interaction model of OxyS-*fhlA *[11]. The prediction was performed (a) without and (b) with extension of the constrained stems. Angle brackets indicate *inter*-molecular base pairs between the two RNAs. Round and square brackets indicate *intra*-molecular base pairs. Square brackets indicate base pairs of the partial structure *σ*^p^. Shown are all columns with < 75% gaps. Jalview [33] was used for alignment visualisation.

Figure 4 shows the alignment and joint secondary structure prediction of the OxyS-*fhlA *complex, *i.e*., the secondary structures of OxyS and *fhlA *and the interaction between them, as predicted by our algorithm. The result of the prediction without extending the constrained stems is shown in Figure 4a, and the result with the extension of the constrained stems is shown in Figure 4b.

For OxyS-*fhlA*, our algorithm was able to consistently predict one of the two interaction sites. The second interaction site, which is situated in the *fhlA *coding region, was only predicted when the constrained stems were not extended in Step 1 of our algorithm. Otherwise the stem of *fhlA *that resides the second interaction site was extended both by inner and outer base pairs. Consequently, the unpaired region of the hairpin containing the second interaction site became shorter such that no interaction was predicted at this site.

### Algorithmic restrictions and potentials

The algorithm supports pseudoknots between the i*ntra*-molecular and *inter*-molecular base pairs, while the time complexity of *O*(*N *× *I *× *L*^3^) is much lower than that of other approaches with the same ability. The time complexity is in the magnitude of PET-fold for the added sequence length *L *of both alignments, and it is linear with respect to the number of sequences *N *in the alignments and the number of iterations *I *in the adaptation of *δ *to find probable partial structures (*I *<*M*/2, where *M *is the sequence length of the longer alignment).

Pfold combines a SCFG with evolutionary information from an alignment of RNA sequences through an explicit evolutionary model. It is not clear whether the model learned from tRNA and rRNA secondary structures is appropriate for RNA cofolding. To avoid the bias of a wrong prior probability distribution Pr[*σ*|*T*, *M*] during the evolutionary scoring of the cofolding step, we omitted all SCFG rule probabilities as well as base pair substitution rates. In these cases, all base pair probabilities were calculated independently, and the different substitution rates of base pairs were ignored; thus, we observed that the entire performance decreased. A possible optimisation would be an adapted prior distribution for the cofolding step, which could be generated when sufficient verified RNA-RNA interaction data becomes available. However, the prediction of RNA secondary structure using evolutionary history is robust for different evolutionary speeds and substitution rate variations [32]. Hence, it is reasonable to assume that the deviation is fairly low using the prior probability distribution of *intra*-molecular structures.

The presented method assumes that both alignments have the same evolutionary history during the cofolding step. A more accurate method would consider independent phylogenetic trees for both RNAs, such as in Step 1, and a common tree for the interaction site. However, we do not know the interacting region in advance; thus, we would need an expectationm maximization algorithm, which would increase the running time of the algorithm to an unreasonable level. Furthermore, the energy contribution of the cofolding step might be slightly biased due to the constraint of the partial structures as single-stranded. We partly solve the resulting *intra*-molecular false predictions by extending the reliable stems in the partial structures, and, as already mentioned above, the RNAcofold algorithm and scoring scheme could be adapted to handle base pair constraints as single-stranded.

Furthermore, there is a limitation of the presented method with regard to interaction sites that are located outside conserved RNA structures. These regions are hard to align if they are, in addition, sequentially unconserved. Thus, our method will most likely miss binding sites located in unstructured and otherwise unrelated regions, *e.g*., miRNA target sites in UTR regions. However, once a correct alignment is found for these regions, then the presented approach still works if the interaction region is conserved or shows enough covariation.

Our algorithm is able to predict pseudoknots between the *intra*-molecular and (inter-)molecular base pairs. In addition, we are interested in more pseudoknots that can be predicted in a similar way using a pipeline of constrained structures. During an iteration of Step 2, additional reliable partial *inter*-molecular structures are constrained as long as new reliable base pairs appear. The final consensus structure is the union of all cofolding base pairs and the partial structures. The main unsolved problem is the weighted combination of the decreasing partial structure probabilities in one scoring scheme when the amount of constraints increases with each iteration.

## Conclusions

In summary, we introduced an extension of the PET-fold algorithm for the identification of interactions between two sets of multiple aligned RNA sequences, which exploits compensating base changes while taking *intra*-molecular partial structures and interaction sites into account. The implementation of the algorithm in PETcofold and its application are described in Seemann *et al*. (submitted).

## Competing interests

The authors declare that they have no competing interests.

## Authors' contributions

SES, RB and JG designed the algorithm. SES implemented the algorithm. ASR designed and performed the analysis of the algorithm. RB drafted the manuscript. All authors contributed to the manuscript, read and approved the final manuscript.

## Supplementary Material

Additional file 1 **Probability distributions of the ****Pfold ****model and Implications of the independence property**. Combined probability distributions of the Pfold model and theimplications of the independence property (Equation (11)) for partial structures are described. Click here for file
