# Supplementary material for: Hierarchical folding of multiple sequence alignments for the prediction of structures and RNA-RNA interactions
Source: Algorithms Mol Biol. 2010 May 21;5:22. doi: 10.1186/1748-7188-5-22 (PMC2903599; doi:10.1186/1748-7188-5-22)
Supplement: Additional file 1 — Probability distributions of the Pfold model and Implications of the independence property. Combined probability distributions of the Pfold model and theimplications of the independence property (Equation (11)) for partial structures are described. [file 1748-7188-5-22-S1.PDF]

Hierarchical folding of multiple sequence alignments for the prediction of structures and RNA-RNA interactions

Stefan E Seemann Andreas S Richter Jan Gorodkin Rolf Backofen

Additional file 1

SCFG for the Pfold model

Let $\mathcal{A}$ be an alignment, and let $\vec{\mathcal{A}}^1, \ldots, \vec{\mathcal{A}}^m$ be the tuple of columns of $\mathcal{A}$, where m is the length of the alignment $\mathcal{A}$, and $\vec{\mathcal{A}}^i$ is the ith column of $\mathcal{A}$. We are interested in the probability distribution of structures $\Pr[\sigma|\mathcal{A}, T, M]$, given the data (i.e., the multiple alignment $\mathcal{A}$ of the sequences $s_1 \ldots s_n$) and the background information (i.e., the secondary structure background model M and the tree T). This is achieved in the Pfold model using a combined SCFG, which gives rise to a combined distribution

$$\Pr[\sigma, \mathcal{A}|T, M] = \Pr[\mathcal{A}|T, \sigma, M] P[\sigma|T, M]$$

Note that $\Pr[\sigma|T, M] = \Pr[\sigma|M]$ does not depend on T and provides an a priori distribution of secondary structures.

The evolutionary model combined with the SCFG is defined as follows. Let $\tau_M(\sigma)$ be the associated parse tree that produces the structure σ using the grammar M. For each node n in $\tau_M(\sigma)$, let label(n) be the associated terminal or non-terminal symbol, rule(n) the associated grammar rule that produced this node, and pos$(n) = (i, j)$ the pair of start and end position of the produced sequence covered by the node (i.e., the leafs below n is the sequence $s_i \ldots s_j$). $\mathcal{A}_{(i,j)}$ denotes the corresponding sub-alignment. Furthermore, let $n_1 \ldots n_k$ be the children of n. Then we recursively define

$$\Pr_{\tau_M(\sigma)}(n, \mathcal{A}_{\mathrm{pos}(n)}) = \left(\prod_{\ell=1}^{k} \Pr_{\tau_M(\sigma)}(n_\ell, \mathcal{A}_{\mathrm{pos}(n_\ell)})\right) \times \Pr[\mathrm{rule}(n)|M] \times \begin{cases} \Pr_{\mathrm{bp}}[\vec{\mathcal{A}}^i \vec{\mathcal{A}}^j|T] & \text{if rule}(n) = F \to dFd \\ & \text{or rule}(n) = L \to dFd \\ \Pr_{\mathrm{sg}}[\vec{\mathcal{A}}^i|T] & \text{if rule}(n) = L \to s \\ 1 & \text{else} \end{cases} \quad (1)$$

where $\Pr_{\mathrm{bp}}[\vec{\mathcal{A}}^i \vec{\mathcal{A}}^j|T]$ and $\Pr_{\mathrm{sg}}[\vec{\mathcal{A}}^i|T]$ are calculated in Pfold using Felsenstein's dynamic programming for phylogenetic trees. The first term of the multiplication in Equation (1) is the recursive decent, the second provides $\Pr[\sigma|M]$ and the last term reflects $\Pr[\mathcal{A}|T, \sigma, M]$. In principle, it is just the recursive definition of the probability of a parse tree given a grammar and extended by position-specific probabilities for producing the terminals. For nodes n that are leaves, we define $\Pr_{\tau_M(\sigma)}(n, \mathcal{A}) = 1$. Finally, we get

$$\Pr[\mathcal{A}, \sigma|T, M] = \Pr_{\tau_M(\sigma)}(\mathrm{r}(\sigma), \mathcal{A}),$$

where $\mathrm{r}(\sigma)$ is the root node of $\tau_M(\sigma)$.

However, we need $\Pr[\sigma|\mathcal{A},T,M]$ for our purpose.By the Bayesian formula we have

$$\Pr[\sigma|\mathcal{A},T,M] = \frac{\Pr[\sigma, A|T,M]}{\Pr[\mathcal{A}|T,M]}.$$

Hence, we need to calculate $\Pr[\mathcal{A}|T,M]$, which we can achieve by marginalisation of σ:

$$\begin{aligned}\Pr[\mathcal{A}|T,M] &= \sum_{\sigma} \Pr[\mathcal{A}|\sigma,T,M] \times \Pr[\sigma|T,M] \\ &= \sum_{\sigma} \Pr[\mathcal{A},\sigma|T,M],\end{aligned}$$

which can be calculated from the SCFG described in Equation (1) by not searching for the parse tree with maximal probability, but by summing over all possible parse trees, which can be done with a DP-like method.

Implications of the Independence Property

The independence property of Equation (11) also indicates the independence properties for the partial structures:

$$\begin{aligned}\Pr[\mathcal{E}(\sigma_1^{\mathrm{p}} \cup \sigma_2^{\mathrm{p}})|s_1\&s_2] &= \sum_{\sigma_1\in\mathcal{E}(\sigma_1^{\mathrm{p}})} \sum_{\sigma_2\in\mathcal{E}(\sigma_2^{\mathrm{p}})} \Pr[\mathcal{E}_{\mathrm{int}}(\sigma_1 \cup \sigma_2)|s_1\&s_2] \\ &\overset{Eq.\,(11)}{=} \sum_{\sigma_1\in\mathcal{E}(\sigma_1^{\mathrm{p}})} \sum_{\sigma_2\in\mathcal{E}(\sigma_2^{\mathrm{p}})} \Pr[\sigma_1|s_1] \times \Pr[\sigma_2|s_2] \\ &= \sum_{\sigma_1 \,\in\, \mathcal{E}(\sigma_1^{\mathrm{p}})} \Pr[\sigma_1|s_1] \times \left(\sum_{\sigma_2 \,\in\, \mathcal{E}(\sigma_2^{\mathrm{p}})} \Pr[\sigma_2|s_2] \right) \\ &= \sum_{\sigma_1 \,\in\, \mathcal{E}(\sigma_1^{\mathrm{p}})} \Pr[\sigma_1|s_1] \times \Pr[\mathcal{E}_2(\sigma_2)|s_2] \\ &= \Pr[\mathcal{E}_2(\sigma_2)|s_2] \times \sum_{\sigma_1 \,\in\, \mathcal{E}(\sigma_1^{\mathrm{p}})} \Pr[\sigma_1|s_1] \\ &= \Pr[\mathcal{E}_1(\sigma_1)|s_2] \times \Pr[\mathcal{E}_2(\sigma_2)|s_2] \qquad (11')\end{aligned}$$
